# Prevalence of transient hyperglycemia and diabetes mellitus in pediatric patients with acute leukemia

**Published:** 2014-03-15

**Authors:** A Banihashem, A Ghasemi, N Ghaemi, N Moazzen, A Amirabadi

**Affiliations:** 1 Associated Professor of Pediatric Hematology & Oncology, faculty of medicine, Mashhad University of Medical Sciences, Mashhad, Iran.; 2 Assistant Professor of Pediatric Hematology & Onc1 Associated Professor of Pediatric Hematology & Oncology, faculty of medicine, Mashhad University of Medical Sciences, Mashhad, Iran.; 3 Associate Professor of Pediatric Endocrinology, Mashhad University of Medical Sciences, Ghaem Hospital, Mashhad, Iran.; 4 Pediatrician, Mashhad University of Medical Sciences, Mashhad, Iran.; 5 Radiation oncologist, cancer research center, Mashhad University of medical sciences, Mashhad, Iran.

**Keywords:** Acute Leukemia, Diabetes Mellitus, Hyperglycemia

## Abstract

**Background:**

The most common malignancy of children is Leukemia, accounting approximately one third of cancer diagnosis. Available data demonstrate improvement in survival of pediatric leukemia, so evaluation of side effects of treatment is very important. This study investigates hyperglycemia and diabetes mellitus prevalence in pediatric patients with acute leukemia.

**Materials and Methods:**

This study was performed in children with acute leukemia. At the first admission, demographic data was collected and blood glucose and HbA1c levels were obtained. These tests examined at least two times during six months of follow up. Growth parameters and blood samples were obtained too.

**Result:**

Twenty nine patients were examined; three of them (10.3%) had diabetes mellitus and 5patients (17.2%) had transient hyperglycemia.Mean age of the patients was 6.26 years and nineteen Children (63%) were in preschool age. In preschool age children, incidence of hyperglycemia was meaningfully higher than school age children (p= 0.02).

24 of 29 patients (82.7%) were known case of acute lymphoblastic leukemia (ALL) and 5 patients (17.3%) were known case of acute myeloblastic leukemia (AML). No significant difference was found between sex groups. Also underweight was significant risk factor for hyperglycemia.

**Conclusion:**

The prevalence of hyperglycemia was in the range of other similar studies in different parts of the world. Underweight and preschool ages were significant predictors of hyperglycemia.

## Introduction

Leukemia is one of the most common malignancies that affect children, accounting for approximately a third of cancer diagnoses (1). It may be defined as a neoplastic disease that involves the blood forming stem cells of the bone marrow, lymph nodes, and spleen. A range of extracellular protein factors regulates the growth and differentiation of cells. This ensures that the mature blood cell types are produced in appropriate proportions.

Acute lymphoblastic leukemia (ALL), the most common malignancy of childhood, accounts for 75% of the leukemia cases, and is curable in 80% to 85% of the patients(2). 

Hyperglycemia occurs in approximately 10 to 16% of patients in remission induction therapy course including a gluoccorticoid and L asparaginase for acute leukemias especially ALL(3-6). Hyperglycemia can be associated with some complications such as non ketotic hyperglycemic hyperosmolar syndrome or diabetic ketoacidosis. Hyperglycemia is associated with increased mortality in critically ill adult and pediatric patients without a previous history of diabetes Mellitus, though no specific level of hyperglycemia has been showed that correlates with the increased morbidity and mortality in these patients (7-9).

In addition to increased mortality rates, more infections have also been noted in patients who experience hyperglycemia during illness (10,,11). However, the prevalence of hyperglycemia either transient or permanenet during remission induction therapy in patients with Acute Leukemia is unknown, and is the subject of this study.

## Materials and Methods

This study was a cross sectional study that consists of children with acute leukemia that were in the first six months of treatment at Dr sheikh children’s hospital in Mashhad university of medical sciences (MUMS). Patient selection was performed with simple sampling. Sample volume was estimated according to similar articles as 25 cases according to estimated 16% incidence of hyperglycemia in leukemic children.Inclusion factors in this study were as below:

- Children with age of under 14 years old in diagnosis 

- Acute leukemia was diagnosed according to bone marrow aspiration, flowcytometry and cytogenetic.

- Evaluation in the first six months of treatment 

And patients with past history of Diabetes mellitus, Central Nervous System involvement and Patients with seizure or sepsis excluded from the study.

The diagnosis of hyperglycemia was when any subjects had at least 2 glucose >140 mg/dL and the patient who had 2 fasting glucose level >126 mg/dL or two random glucose level >200 mg/dL named as a diabetic patient. 

The patients with hemoglobin level under 8 mg/dl named as anemic and others as normal.

The patients included in this study were those who underwent chemotherapy by the authors in the Dr sheikh children’s hospital. This report consisted of 29 consecutive patients who were treated with chemotherapy.

At first, patients examined by a pediatrician. Height, weight and BMI of patients were assessed three times and family history of them was records. Fasting or random blood glucose and HbA1c were measured and patients whose blood glucose level were in the range of diabetes examined by a pediatrics endocrinologist. CBC, fasting or random blood glucose and HbA1c assessed three months and six months after starting treatment. After six months the patients were examined again and the results evaluated. 

Final relation of hyperglycemia with above parameters was assessed and the role of several factors such as age, sex, anemia, fasting or random blood glucose, HbA1c, use of different chemotherapy regimens, history of diabetes and other factors was evaluated.

Data was analyzed by SPSS software (Ver. 18) and Chi-square test was used to compare failure rate between groups. P values < 0.05 were regarded as significant.

## Results

Thirty two patients with a diagnosis of Acute Leukemia were evaluated at least three times during the study period. Three patients had a diagnosis of Acute Leukemia, but no complete information was available. .Finally 29 patients evaluated for hyperglycemia and diabetes mellitus. 

Patients were children with age range of 1 to 14 years old (mean age = 6.26 years old). We divide the patients into two categories. The cases that were 6 and under 6 years old 65.5% (19 out of 29 subjects) called preschool and the others 34.5% of subjects (10 out of 29 subjects) called school age. 

When compare the result of diabetes to age groups, significant correlation was observed between age and diabetes mellitus incidence. (Chi-square p=0.02) (Table 1).

In according to sex categories the cases 51.7% were male (15 out of 29 subjects) and 48.2% were female (14 out of 29 subjects). No significant difference was detected in the incidence of diabetes mellitus and hyperglycemia between sex groups (chi-square p=0.654).

In addition, times of admission were more than 4.7 admissions during the study period with an average length of hospitalization more than 11 days. In this study, There were 82.7% known case of acute lymphoblastic leukemia (24 out of 29 subjects) and the remainder were known case of acute myeloblastic leukemia (5 out of 29 – 17.3%).There was no significant difference between incidence of diabetes mellitus and hyperglycemia and the type of the acute leukemia (chi-square p=0.654).

In according to above definition 10.3% of patients had diabetic (3 out of 29 subjects) and 17.2% of patients had hyperglycemia in one sampling (5 out of 29 subjects) but that did not repeat in next sampling. So we didn't add them to diabetic patient.

The patients were evaluated about their blood count parameters such as hemoglobin levels. The patients were divided to two groups. In according to this definition 37.9% (11 out of 29 subjects) were anemic and 81.9% (18 out of 29 subjects) were normal hemoglobin level. 

There was no significant difference between incidence of diabetes mellitus and hyperglycemia between type of the acute leukemia (chi-square p=0.573).

**Figure 1:**
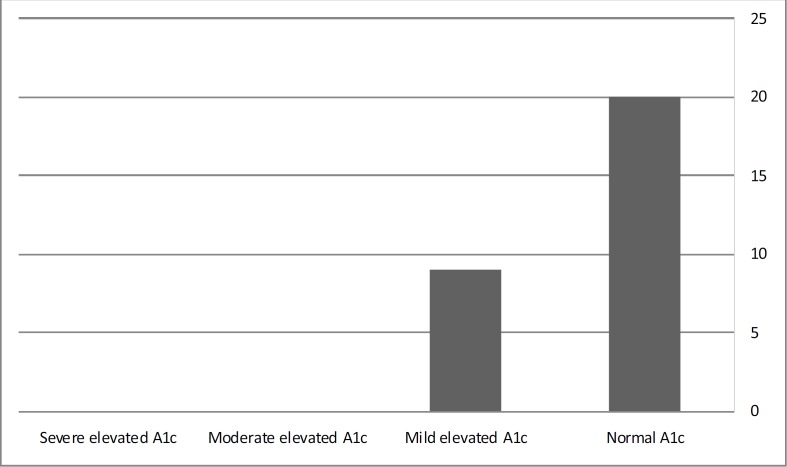
HbA1C Levels in patients (Severe, moderate, mild and normal A1C)

In 34.5% of patients (10 out of 29 subjects) there were familial history of diabetes mellitus in their first degree relatives and others 65.5% of subjects (18 out of 29 subjects) did not had such history, No significant difference was detected in incidence of diabetes mellitus and hyperglycemia between sex groups (chi-square p=0.654). According to this study incidence of diabetes mellitus and hyperglycemia was higher in children without familial history of diabetes mellitus. The hemoglobin A1C of all patients was evaluated in each glucose level assessment. The patients were divided to four categories. When A1C level were under 6% it was defined as normal, when between 6% to7.99% called mild elevation. The levels of 8 to 9.99% were moderate and the A1C levels were 10% or above called severe elevated. In this study, there was any patients with moderate or severe A1C levels (Figure 1). The significant correlation was not observed between HbA1c levels and diabetes mellitus and hyperglycemia incidence. (Chi-square p=0.386). In according to Body mass index for age percentiles, all of the patients categorized to four groups. All patients with BMI of fewer than 5 percentile named underweight.

The cases with BMI between 5 and 85 named normal weights and BMI between 85 and 95 named overweight and above 95 were obese. 

The significant reverse correlation was observed between diabetes mellitus and hyperglycemia incidence and BMI score. (Chi-square p=2.045). 

Treatment protocols of the patients were different. Their chemotherapy regimens were UKALL version 9, A, B and C. There was no significant difference between incidence of diabetes mellitus and hyperglycemia between different protocol of chemotherapy that used for treatment of children (chi-square p=0.983). In patients who had diabetes mellitus, the level of insulin, c-peptide, cortisol, GAD (glutamic acid decarboxylase) , ICA(islet cell auto antibodies) was evaluated and they examined for autoimmunity or other related hyperglycemia due to excess hormones alone or due to puberty . All of them were in normal values and it didn’t find autoimmunity or prolonged hyperglycemia (normal HbA1C). This data was showed in Table 2.

**Tabel I:** Comparison between age groups and Diabetes Mellitus in paients with leukemia

			**Diabetes**		**Total**
			Negative	Positive	
**Age**	6and below	Count	17	2	19
		% Within Age	89.5%	10.5%	100.0%
	Over 7	Count	9	1	10
		% Within Age	90.0%	10.0%	100.0%
**Total **		Count	26	3	29
		% Within Age	89.7%	10.3%	100.0%

**Table 2:** Hormonal assessment (level of insulin, c-peptide, cortisol, GAD, ICA) and puberty stage

**HbA1c**	**Insulin**	**c-peptide**	**cortisole**	**GAD**	**ICA**	**Puberty stage**
**5.9%**	51 mg/dl	4.9 mg/dl	14 mg/dl	3.2	0.7	1 tanner
**4.7%**	8 mg/dl	0.52 mg/dl	16 mg/dl	212	2.1	2 tanner
**5.9%**	49 mg/dl	4 mg/dl	12 mg/dl	3	1.1	1 tanner

## Discussion

In this study, 29 children with diagnosis of acute leukemia that treated in Dr sheikh children’s hospital and were in the first six months of their treatment, evaluated for hyperglycemia. These Children followed for six months and at intervals of three months were assessed for growth parameters, blood sugar and HbA1c and other possible risk factors of hyperglycemia. Hyperglycemia defined when at least two glucose >140 mg/dl and the patient who had two fasting glucose level >126 mg/dl or two random glucose level >200 mg/dl named as a diabetic patient. According to these criteria, there were three cases with diabetes mellitus (10.3% of the subjects).

Patients were children in the range of 1 to 14 years old (mean age = 6.26 years old). In according to sex categories the cases were 51.7% male (15 out of 29 subjects) and 48.2% female (14 out of 29 subjects). There were 82.7% known case of acute lymphoblastic leukemia (24 out of 29 subjects) and the remainder were known case of acute myeloblastic leukemia (5 out of 29 = 17.3%).In according to above definition 10.3% subjects had diabetic (3 out of 29 subjects).

According to laboratory reference value, among these patients with hyperglycemia, two girls were 4 and 3.5 years old and one boy was 14 years old. All of them had ALL and neither of them have had family history of diabetes mellitus. Two of them treated with UKALL-A regimen and one of them treated with UKALL-b. They need to insulin therapy for their hyperglycemia, but their hyperglycemias were not permanent and after a short period of follow up they were euglycemic and in follow up they had not any typical symptoms for hyperglycemia. In addition, they evaluated by blood glucose after that in their routine follow up for their disease but it weren’t recurring.

The most similar study was a study by Baillargeon et al. that evaluated transient hyperglycemia in Hispanic children with Acute Lymphoblastic Leukemia. Transient hyperglycemia occurs commonly during the treatment of childhood acute lymphoblastic leukemia (ALL). The purpose of Baillargeon study was to examine the incidence of and risk factors for transient hyperglycemia during induction chemotherapy in Hispanic pediatric patients diagnosed with B-Precursor ALL. The study cohort consisted of 155 Hispanic pediatric patients diagnosed with ALL and treated at South Texas pediatric oncology centers between 1993 and 2002. Overall, 11.0% of the study cohort developed transient hyperglycemia during induction chemotherapy. Age and body mass index (BMI) were both positively associated with the risk of hyperglycemia. Females exhibited a substantially higher risk of hyperglycemia than males, but this association did not reach statistical significance after adjusting for other covariates. Among patients who developed hyperglycemia, 100% of those who required insulin were in the 13 to 18-year age group and reported a family history of diabetes. Hyperglycemic patients classified as obese (BMI ≥ 95 centile) were more than twice as likely to have required insulin therapy compared to overweight patients (BMI 85–<95 centile) and three times as likely to have required insulin compared to normal weight (BMI <85 centile) patients.The incidence of chemotherapy-induced transient hyperglycemia in the present study cohort was comparable to that reported in previous pediatric ALL patients. 12When compare the result of this study to these findings, the incidence of hyperglycemia was relatively equal, but there was no association between BMI or age with hyperglycemia.

In a study by Tamez Perez et al. glucose disturbances in non-diabetic patients receiving acute treatment with methylprednisolone pulses was assessed. They found no correlation between the magnitude of hyperglycemia and the studied variables.Methylprednisolone pulses produced significant increases in fasting glucose in most patients without diabetes(13).

Diabetic ketoacidosis during therapy for pediatric acute lymphoblastic leukemia was title of a study by Roberson et al. Hyperglycemia is common during therapy for acute lymphoblastic leukemia (ALL), but diabetic ketoacidosis (DKA) occurs rarely (14).

At a literatue by Lowas et al. Prevalence of transient hyperglycemia during induction chemotherapy for pediatric acute lymphoblastic leukemia was assessed. Transient hyperglycemia (TH) is a recognized side effect of the corticosteroids and asparaginase given during induction chemotherapy for pediatric acute lymphoblastic leukemia (ALL). Information is needed about how TH has been impacted by changes in ALL therapy. This study examined the prevalence of TH in a cohort of pediatric ALL patients and the impact on TH of type of steroid or asparaginase used and another risk factors for hyperglycemia such as age, gender, and overweight. Retrospective record review of patients aged 2-18 years diagnosed with ALL in 1999-2006. TH was defined as >or=2 random glucose values >or=200 mg/dl. Overall prevalence of TH was calculated. Risk factors were evaluated using Chi-square analysis and logistic regression. One hundred sixty-two subjects (70 female) were reviewed, 33 (20.4%) of whom had TH. 42.2% of subjects over age 10 years had TH, compared to 12.0% of younger children (P < 0.001). No gender difference was found. Overweight (BMI >or= 95th percentile) and at risk for overweight (BMI >or= 85th percentile) were significant risk factors for TH (P = 0.007 and P = 0.003, respectively). Native L-asparaginase was associated with increased TH compared to PEG-asparaginase (P = 0.047). There was a non-significant trend to TH in patients who received prednisone, but this disappeared in multivariate analysis. The prevalence of TH in this study was higher than previously reports. Overweight, age >or=10 years, and use of native L-asparaginase were significant predictors of TH.15In contrast to this study, its found that preschool age was significant predictor of hyperglycemia. The data showed that there was no correlation between BMI score and hyperglycemia incidence. 

## Conclusion

The prevalence of hyperglycemia was in the range of similar studies other where in the world. 10.3% of patients had diabetes mellitus and 5patients (17.2%) had transient hyperglycemia Underweight and preschool ages were significant predictors of hyperglycemia.

## Conflict of interest

The authors have no conflict of interest.
